# Cellular microRNA let-7c inhibits M1 protein expression of the H1N1 influenza A virus in infected human lung epithelial cells

**DOI:** 10.1111/j.1582-4934.2012.01572.x

**Published:** 2012-09-26

**Authors:** Yong-Jie Ma, Jing Yang, Xing-Liang Fan, Hai-Bao Zhao, Wei Hu, Zhen-Peng Li, Guang-Chuang Yu, Xiao-Ran Ding, Jun-Zhi Wang, Xiao-Chen Bo, Xiao-Fei Zheng, Zhe Zhou, Sheng-Qi Wang

**Affiliations:** a Lab of Biotechnology, Beijing Institute of Radiation MedicineBeijing, China; b Astronaut Research and Training Center of ChinaBeijing, China; c Department of Biopharmaceutics School of Pharmacy, Fourth Military Medical UniversityXi'an, China

**Keywords:** influenza virus, microRNA, epithelial

## Abstract

The influenza virus (IV) triggers a series of signalling events inside host cells and induces complex cellular responses. Studies have suggested that host factors play an essential role in IV replication. MicroRNAs (miRNAs) represent a class of small non-coding RNAs that target mRNAs, triggering either translation repression or RNA degradation. Emerging research suggests that host-derived cellular miRNAs are involved in mediating the host–IV interaction. Using miRNA microarrays, we identified several miRNAs aberrantly expressed in IV-infected human lung epithelial cells (A549). Specifically, miR-let-7c was highly up-regulated in IV-infected A549 cells. PITA and miRanda database screening indicated that the let-7c seed sequence is a perfect complementary sequence match to the 3′ untranslated region (UTR) of viral gene M1 (+) cRNA, but not to PB2 and PA. As detected by a luciferase reporter system, let-7c directly targeted the 3′-UTR of M1 (+) cRNA, but not PB2 and PA. To experimentally identify the function of cellular let-7c, precursor let-7c was transfected into A549 cells. Let-7c down-regulated IV M1 expression at both the (+) cRNA and protein levels. Furthermore, transfection with a let-7c inhibitor enhanced the expression of M1. Therefore, let-7c may reduce IV replication by degrading M1 (+) cRNA. This is the first report indicating that cellular miRNA regulates IV replication through the degradation of viral gene (+) cRNA by matching the 3′-UTR of the viral cRNA. These findings suggest that let-7c plays a role in protecting host cells from the virus in addition to its known cellular functions.

## Introduction

Influenza A viruses (IAVs) can infect a broad spectrum of hosts (birds, humans and other mammals) and evolve into highly pathogenic strains [1, 2]. Influenza A virus is an RNA virus that encodes up to 11 proteins. The small coding capacity demands that the virus exploits the host's cellular factors for many vital aspects of its life cycle. Influenza A viruses are among the most common causes of human respiratory infections and are associated with high morbidity and mortality. Previous studies indicated that many mammalian host proteins mediate the IAV-induced host response. Moreover, these protein–protein interactions between virus and host are critical for viral propagation. Furthermore, efficient influenza virus propagation critically depends upon the activation of host caspase-3, as the presence of a caspase-3 inhibitor in cells strongly impairs viral replication. In the present study, caspase-3 was partially knocked down by small interfering RNAs [3]. Turpin *et al*. showed that p53 levels and activity were up-regulated during influenza infection, whereas inhibition of p53 led to increased viral titres [4]. Recently, cyclophilin A was found to interact with the IAV-encoded M1 protein and impair early viral replication [5]. Based on a genome-wide RNA interference screening, Konig *et al*. identified 295 cellular cofactors required for early stage replication of the influenza virus [6].

MicroRNAs are single-stranded RNA molecules that are 21–23 nucleotides in length [7]. Several hundred miRNAs have been identified in plants, animals and viral RNA genomes [8, 9]. In animals, miRNAs regulate many cellular processes by binding to 3′-UTRs of their target mRNAs, causing translational repression of the target mRNA [8]. Recent studies showed that viral miRNAs play an important role in regulating viral infection in host cells by targeting the cellular or viral genes. MicroRNAs encoded by Simian virus 40 (SV40) were reported to target their own virally encoded T antigen, leading to a decrease in cytotoxic T lymphocyte-mediated lysis of infected cells [10]. miR-latency-associated transcript (LAT), an miRNA produced from the viral LAT of herpes simplex virus type 1, targets the cellular mRNAs encoding transforming growth factor β and SMAD3 that results in inhibition of cell proliferation and apoptosis [11]. These two examples clearly indicate that viral miRNAs are capable of controlling expression of viral or cellular genes.

Conversely, cellular miRNAs can regulate viral infections. Host-derived miR-24 and miR-93 have been found to target the viral large protein (L protein) and phosphoprotein (P protein) genes, respectively. A deficiency in miR-24 and miR-93 was responsible for increased vesicular stomatitis virus (VSV) propagation in Dicer1 knockout cells [12]. Furthermore, Lecellier *et al*. reported that host miR-32 effectively inhibits the accumulation of the retrovirus primate foamy virus type 1 (PFV-1) in human cells by targeting a sequence in the genome of the PFV-1 [13]. Huang *et al*. reported that a cluster of cellular miRNAs, including miR-28, miR-125b, miR-150, miR-223 and miR-382, targets the 3′ ends of various human immunodeficiency virus 1 (HIV-1) mRNAs. These host miRNAs are enriched in resting CD4^+^ T cells as compared to activated CD4^+^ T cells, indicating that these cellular miRNAs are important to maintain HIV-1 latency [14]. Inhibition of miR-122, a cellular miRNA highly and specifically expressed in the human liver, resulted in a marked loss in autonomous replication of hepatitis C viral RNAs, suggesting that miR-122 likely enhances propagation of the virus [15, 16].

Most recently, Li *et al*. reported that cellular miRNA expression could be altered during influenza virus infection, irrespective of the lethality of the virus [17]. In the present study, a miRNA array was used to screen and identify possible cellular miRNAs involved in regulating influenza virus infection in human lung epithelial cells; let-7c was identified to specifically interact with IAV matrix protein (M1) (+) cRNA. Functional analysis indicated that let-7c inhibited the replication of the virus in human cells. As M1 is the most abundant protein in the IAV viral particle [18], and M1 mutation studies indicate that M1 is involved in virus–host protein interactions [19], the findings of the present study suggest that cellular let-7c may act as a potential therapeutic target to inhibit influenza virus infection through its direct interaction with the viral M1 mRNA.

## Materials and methods

### Virus and cells

Influenza A/JingFang/86-1(H1N1) virus or influenza A/FM/1/47 (H1N1) virus was grown in the allantoic cavity of 10-day-old embryonated chicken eggs (Specific Pathogen Free, Merial-Vital Laboratory Animal Technology, Beijing, China) for 48 hrs at 35°C. The allantoic fluid was clarified by centrifugation at 6000 × *g* for 15 min. and stored at −80°C until use. The experiments were reviewed and approved by the Animal Ethics Committee of the Beijing Institute of Radiation Medicine in accordance with the regulations of Beijing Administration Office of Laboratory Animal (No. SCXK-BJ-2009-0003). Virus production was determined by measuring haemagglutinin units. Human lung epithelial cells (A549) were purchased from the American Type Culture Collection (ATCC) and cultured in F12K medium containing 10% foetal bovine serum (FBS) with 50 U/ml gentamicin at 37°C with 5% CO_2_. Madin-Darby canine kidney (MDCK) cells were kindly provided by the Institute of Virus, Center of Disease Control of China and cultured in Dulbecco's modified Eagle's medium (DMEM) containing 10% FBS.

### miRNA profile

RNA from uninfected and IAV-infected A549 cells was isolated 24 hrs post-infection using the mirVana miRNA Isolation Kit (Ambion, Austin, TX, USA) and miRNA profiles were obtained by Paraflo™ MicroRNA Microarray Assay (LC Sciences, Houston, TX, USA).

### Bioinformatic analysis

The PITA (http://genie.weizmann.ac.il/pubs/mir07) database and miRanda software were used to identify potential targets of let-7c as described previously [20, 21].

### Viral infections

Host cells were washed with phosphate-buffered saline and infected with influenza virus at a multiplicity of infection of 5 for 1 hr at 37°C. After infection, the inocula were removed, and cells were incubated with F12K containing 0.2% bovine serum albumin (BSA) (Gibco, Invitrogen Incorporated, Carlsbad, CA, USA) for the time indicated.

### Cell viability assay

The A549 cells were seeded in 48-well plates. After overnight incubation in a 5% CO_2_ incubator at 37°C, cells were transfected with the miRNA expression vectors or an empty vector and infected with influenza A/JingFang/86-1(H1N1) virus or influenza virus A/FM/1/47 (H1N1) 24 hrs later. Each transfection was performed at least in triplicate. Cell viabilities were assessed by Cell Counting Kit-8 (CCK-8) assay (Dojindo, Shanghai, China) 24 hrs post-infection.

### Viral titres

To measure viral titres, transfected A549 cells were infected with IAV for 1 hr at 37°C. After a 1-hr adsorption, the inocula were removed, and cells were incubated with DMEM containing 0.2% BSA for the indicated times. The virus titres from culture supernatants of A549 cells were determined by measuring 50% Tissue Culture Infective Dose (TCID_50_) in MDCK cells. The titres were evaluated by the method described by Reed and Muench [22].

### qRT-PCR for let-7c and M1 and nucleoprotein viral RNA

To determine the expression of let-7c, Hairpin-it™ Assay kit (GenePharma, Shanghai, China) was used according to the manufacturer's protocol. M1 viral RNA was determined by a TaqMan expression assay (forward primer: 5′-GACCRATCCTGTCACCTCTGAC-3′; backward primer: 5′-AGGGCATTYTGGACAAAKCGTCTA-3′; and probe: FAM-5′-TGCAGTCCTCGCTCACTGGGCACG-3′-TEMRA). Nucleoprotein (NP) viral RNA was determined using a SYBR assay (forward primer: 5′-GCAGAAATCATAAGGATGA-3′; backward primer: 5′-TGTCTCCGAAGAAATAAGA-3′). All RNA determinations were assayed in duplicate and repeated three times.

### Vector construction and transfection

Genomic fragments of *Homo sapiens* let-7a, let-7c, let-7d, mir-7-1, mir-26b, mir-146b and mir-192 precursors were amplified by PCR from A549 cells. The PCR product was cloned into pcDNA3.1(+) (Invitrogen, Carlsbad, CA, USA) digested by *Hin*dIII and *Xba*I. The 3′-UTR segment of influenza virus M1 was amplified by PCR from H1N1 viral cDNA and subcloned into the pMIR-Report luciferase reporter vector (Ambion) digested by *Hin*dIII and *Spe*I. The 3′-UTR segment of influenza virus PA, PB1 and PB2 was synthesized directly. The annealed oligonucleotides were used to ligate into the *Hin*dIII and *Spe*I sites of the pMIR-Report luciferase. Vector transfection was performed with FuGene® HD transfection reagent (Roche, Indianapolis, IN, USA) according to the manufacturer's protocol.

### siRNAs and transfection

The let-7c inhibitor was synthesized by GenePharma. siRNA transfection was performed using Lipofectamine 2000 (Invitrogen). In brief, cells were first plated in a six-well plate to 60% confluence, and 0.4 μM siRNA was then dissolved in 2% Lipofectamine 2000/Opti-MEM medium (Invitrogen) and added into each well. The cells were incubated with the mixture for 6 hrs before replacing the medium. Total RNAs and proteins were prepared 48 hrs after transfection and were used for qRT-PCR and western blotting analysis.

### Western blot analysis

Cell fractions were prepared using M-PER Mammalian Protein Extraction Reagent based on the manufacturer's instruction (Pierce, Rockford, IL, USA). Western blot analysis was performed as described previously [17].

### Luciferase reporter assay

The A549 cells were seeded at 20,000 cells per well in 24-well plates and cotransfected with 100 ng of firefly luciferase reporter vector containing the influenza M1 (+) cRNA 3′-UTR and 8 ng of the β-gal Control Plasmid (Ambion) in a final volume of 0.5 ml using Lipofectamine 2000 (Invitrogen). Forty-eight hours after transfection, both luciferase and β-gal activity were measured and the relative level of luciferase was normalized against the β-gal readings (Promega, Madison, WI, USA).

## Results

### miRNA expression profile analysis of IAV-infected human cells

To determine the ability of IAV (JingFang/86-1(H1N1) virus) to modulate the expression of cellular miRNAs involved in its replication, the expression profile of cellular miRNA in infected and uninfected A549 human lung epithelial cells was compared by microarray analysis (Table 1). Twenty-eight miRNAs were found to be up-regulated or down-regulated upon infection.

**Table 1:** List of miRNAs found to be differentially regulated under influenza virus infection in microarray analysis

miRNA	Fold of control	

	4 hrs after infection	24 hrs after infection
hsa-miR-768-5p	1.49	2.25
hsa-miR-224	2.32	3.20
hsa-miR-30a-3p	1.99	2.61
hsa-let-7c	1.53	1.68
hsa-miR-23b	1.28	1.34
hsa-let-7b	1.67	1.89
hsa-let-7a	1.40	1.49
hsa-let-7e	1.70	1.86
hsa-miR-23a	1.38	1.43
hsa-let-7d	1.87	1.97
hsa-miR-26b	3.16	3.33
hsa-miR-182	1.48	1.50
hsa-miR-34a	2.69	2.77
hsa-miR-26a	2.07	2.08
hsa-miR-98	3.55	3.58
hsa-miR-361	1.67	1.66
hsa-miR-10a	4.00	3.88
hsa-miR-452	2.12	2.07
hsa-miR-29b	3.87	1.40
hsa-miR-15a	8.07	2.57
hsa-miR-374	15.9	4.62
hsa-miR-146a	1.36	0.131
hsa-miR-671	1.46	0.477
hsa-miR-134	1.21	0.744
hsa-miR-7	2.16	0.675
hsa-miR-193b	0.532	0.670
hsa-miR-146b	3.62	0.288
hsa-miR-130	1.10	0.661

### Effect of miRNA overexpression on the viability of IAV-infected A549 cells

**Fig 1:**
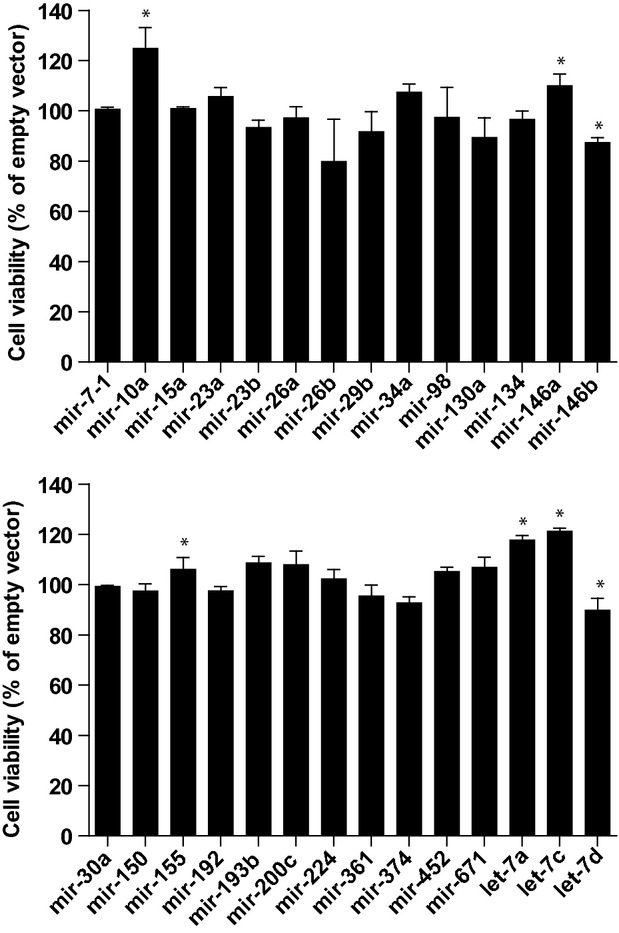
Cell viability after miRNA expression vector transfection in A549 cells. A549 cells were transfected with different miRNA expression vectors and influenza A/JingFang/86-1(H1N1) virus was inoculated 24 hrs later. Twenty-four hours after the inoculation, cell viabilities were assessed by the CCK-8 assay. Data are expressed as mean ± S.E., *n* = 4, *P < 0.05.

Based on the profile screening results, miRNA-expressing vectors were constructed for each of the 28 identified miRNAs. A549 cells were then transfected with each of these miRNA expression vectors or the control empty vector followed by influenza A/JingFang/86-1(H1N1) virus infection 24 hrs later. Cell viability was assessed 24 hrs post-infection. Five miRNAs (miR-10a, miR-146a, let-7a and let-7c) significantly increased cell viability as compared to the cells treated with the control empty vector, whereas two miRNAs (miR-7d and miR-146b) decreased cell viability (Fig. 1).

### miRNA overexpression inhibited IAV titres in infected A549 cells

**Fig 2:**
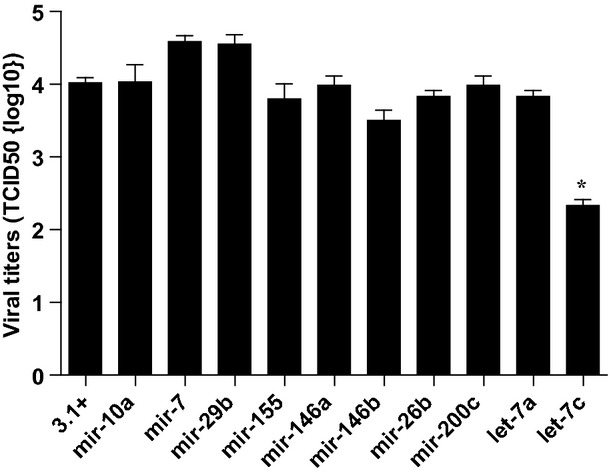
Viral titres in the supernatants of A549 cells after transfection of miRNA expression vectors. A549 cells were transfected with different miRNA expression vectors and influenza A/JingFang/86-1(H1N1) virus was inoculated 24 hrs later. Viral titres were assessed 48 hrs after inoculation. Data are expressed as mean ± S.E., *n* = 4, *P < 0.05.

Viral titres were examined in the infected A549 cells after transfection with the miRNA expression vectors that significantly affected cell viability. The supernatants of influenza A/JingFang/86-1(H1N1) virus-infected cells were collected and transferred onto MDCK cells; viral titres were determined by evaluating the TCID_50_ of the MDCK cells. Among all the miRNA expression vectors that significantly affected cell viability, only the let-7c expression vector significantly decreased viral titres by 50% in comparison to the control vector (Fig. 2).

### Let-7c overexpression inhibited M1 vRNA synthesis

**Fig 3:**
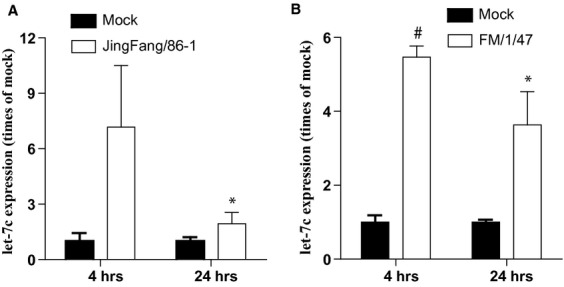
Quantitative real-time PCR analysis of let-7c in influenza virus-infected A549 cells. RNA was isolated from A549 cells after infection with Influenza A/JingFang/86-1(H1N1) virus (A) or influenza A/FM/1/47 (H1N1) virus (B) for 4 or 24 hrs. Let-7c was detected and presented relative to GAPDH mRNA. Data are expressed as mean ± S.E., *n* = 3, *P < 0.05, #*P* < 0.01.

**Fig 4:**
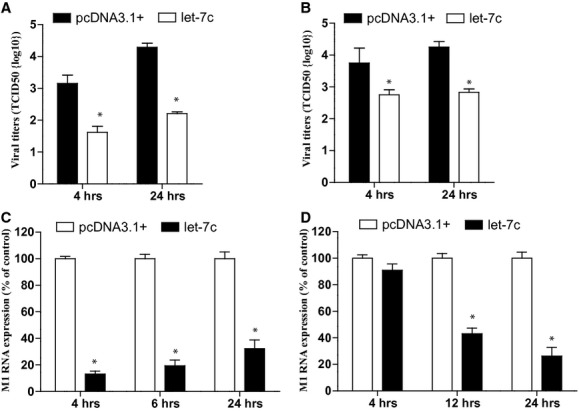
M1 vRNA expression and influenza A virus titres after let-7c overexpression in A549 cells. Twenty-four hours after transfection with let-7c expression vector, A549 cells were infected with influenza A/JingFang/86-1 (H1N1) virus or influenza A/FM/1/47 (H1N1) virus. (A) Influenza A/JingFang/86-1 (H1N1) virus titres in the medium of A549 cells were detected at the indicated time points. (B) Influenza A/FM/1/47 (H1N1) virus titres in the medium of A549 cells were detected at the indicated time points. (C) M1 and NP vRNA levels were determined at 4, 6 and 24 hrs after influenza A/JingFang/86-1 (H1N1) virus infection by q-PCR. (D) M1 and NP vRNA levels were determined at 4, 6 and 24 hrs after influenza A/FM/1/47 (H1N1) virus infection by q-PCR. Data are expressed as mean ± S.E., *n* = 4, *P < 0.01.

Providing further support that host let-7c played a role in regulating IAV infection, let-7c was significantly up-regulated in infected A549 over uninfected A549 cells as measured by the expression of mature let-7c transcript using real-time PCR (Fig. 3). To explore whether or not overexpression of let-7c had any effect on vRNA synthesis, vRNA levels were evaluated in H1N1 virus-infected A549 cells transfected with miRNA vectors (Fig. 4A and B). The quantity of M1 vRNA was analysed at 4, 6 and 24 hrs after infection. As shown in Fig. 4C and D, the level of M1 vRNA was reduced in the cells overexpressing let-7c compared with empty vector-transfected cells. Specifically, an approximate 87% reduction in M1 vRNA at 4 hrs was observed in influenza A/JingFang/86-1(H1N1) virus-infected A549 cells.

### Let-7c interacts with the 3′-UTR of M1

**Fig 5:**
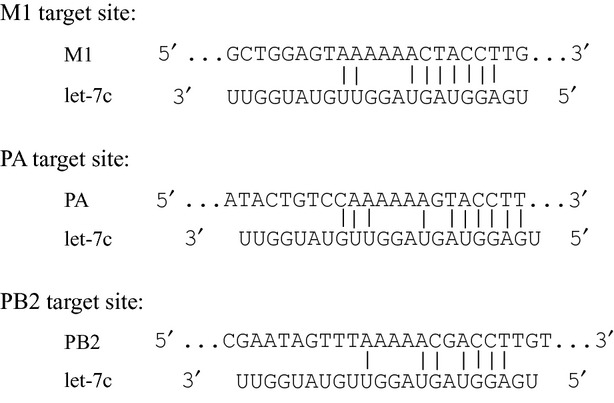
Let-7c target site prediction in the vRNA of influenza virus A Let-7c was predicted to pair with residues in the 3′ region of cRNA of M1, PA and PB2 by PITA and miRanda database screens.

**Fig 6:**
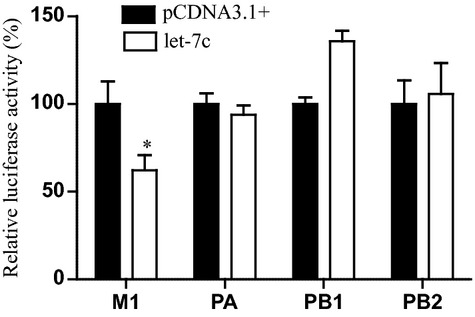
Let-7c target analysis using luciferase reporter assays in A549 cells Luciferase reporter assays of A549 cells 48 hrs after cotransfection of the influenza virus M1, PA, PB1 and PB2 3′-UTR reporter constructs and let-7c expression vector. Data were normalized to β-galactosidase activity. Data are expressed as mean ± S.E., *n* = 3, *P < 0.05.

The PITA and miRanda databases were screened to identify potential let-7c target genes. miRanda indicated that let-7c could directly target the 3′-UTR of viral M1, PA and PB2 (+) cRNA (Fig. 5). The M1, PA and PB2 3′-UTRs were then each cloned into a pMIR-Report™ luciferase reporter vector. The reporter vector was cotransfected with let-7c expression vector or control plasmid into A549 cells. Luciferase activity detection showed that pcDNA3.1(+)/let-7c produced a 40% decrease in pMIR-M1 relative luciferase activity compared with control pcDNA3.1(+)-transfected cells (Fig. 6). No decrease in relative luciferase activity was observed in pMIR-PA, pMIR-PB1 or pMIR-PB2. The above results indicated that let-7c directly targeted the 3′-UTR of M1 (+) cRNA, but not the 3′-UTR of PA or PB2.

### Let-7c regulated the expression of M1 vRNA and protein

**Fig 7:**
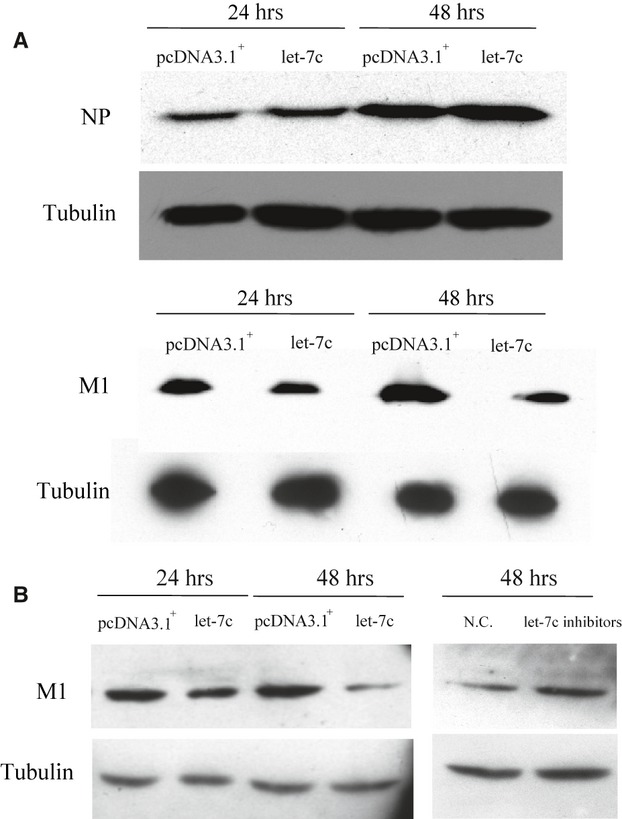
M1 expression after let-7c overexpression in A549 cells. (A) A549 cells were infected with influenza A/FM/1/47 (H1N1) virus for 24 or 48 hrs. Immunoblot analysis for M1 and NP protein in isolated A549 cell protein homogenate was performed. (B) Twenty-four hours after transfection with let-7c expression vector or with a let-7c inhibitor, A549 cells were infected with Influenza A/JingFang/86-1(H1N1) virus for 24 or 48 hrs. Immunoblot analysis for M1 and NP protein in isolated A549 cell protein homogenate was performed. Data shown are representative of three independent experiments.

To further explore whether let-7c could directly regulate M1 protein expression, the effect of let-7c expression vector and let-7c inhibitors on the expression of M1 protein in influenza A/JingFang/86-1(H1N1) virus or influenza A/FM/1/47 (H1N1) virus-infected A549 cells was investigated. Western blot assay indicated that M1 protein was dramatically reduced after pcDNA3.1(+)/let-7c transfection, whereas the control pcDNA3.1(+) vector did not significantly affect M1 protein expression (Fig. 7A). As shown in Fig. 7B, transfection with a let-7c inhibitor enhanced M1 expression as compared to transfection with vehicle alone [23].

## Discussion

In the present study, we indicate that the expression of let-7c is up-regulated in IAV-infected human lung epithelial cells and that overexpression of let-7c results in the inhibition of IAV replication in host cells. This study provides a better understanding of the interaction between IAV and host cells.

Matrix protein (M1) is the most abundant conservative protein in the viral particle. It is a multifunctional protein that plays a role in influenza virus replication and consists of 252 amino acids that are relatively conserved among influenza subtypes [18]. A number of published reports have shown that several host cell factors may be required for regulation of influenza virus replication through interaction with M1 protein during different stages of infection. Viral ribonucleoprotein (RNP)/M1 and NP in the cytoplasm were reported to associate with host cytoskeletal components that may help the viral morphogenesis [24, 25]. Watanabe *et al*. indicated that heat shock cognate protein 70 (Hsc70) was involved in the influenza virus life cycle by interacting with M1 [26]. Intracellular caspase-8 was also found to bind M1 to trigger the caspase-8-mediated apoptosis pathway in influenza virus-infected cells [27, 28]. Furthermore, the cellular receptor of activated C kinase 1 is able to interact with and phosphorylate M1. In the present study, we observed that host cells can also regulate influenza virus replication at the transcriptional level, as human cellular miR-let-7c were shown to degrade M1 (+) cRNA by binding to its 3′-UTR. Therefore, miR-let-7c mediates anti-IAV activity in the lung epithelial cells, and the 3′-UTR terminal sequences of the M1 (+) cRNA can be used as an effective target against viral replication. Furthermore, we indicate that miR-let-7c is another host factor involved in the replication of IAV. The present data suggest that up-regulation of let-7c in lung epithelial cells upon IAV infection may be considered as a self-protective mechanism mediated by the host cells during the infection.

The exact function of let-7 in human development and normal physiology is not yet obvious. Two members of the let-7 family, let-7a and let-7c, have been shown to regulate cell proliferation and to inhibit expression of the oncogenes ras [29] and c-myc [30]. As the influenza virus life cycle involves a large number of cellular proteins and biological pathways, we postulate that let-7c may indirectly affect the influenza virus propagation by directly targeting host protein transcripts as well. Therefore, bioinformatic analysis was performed to identify the host protein targets of let-7c. Let-7c was shown to bind to 3′-UTR residues of the EIF2AK2, MCM5, PA2G4 and caspase-8 host mRNAs (Supplementary 1). Although a trend towards reduction was observed, pcDNA3.1(+)/let-7c did not produce a significant reduction of EIF2AK2, MCM5, PA2G4 or caspase-8 as measured by the luciferase reporter system (Supplementary Figs 2–3). Thus, it is still worthwhile to investigate whether or not let-7c also mediates the virus–host interaction by acting on host proteins/pathways.

Several recent reports have investigated whether or not cellular microRNA is involved in influenza virus infection. Li *et al*. reported that cellular miRNA expression is altered during influenza virus infection, irrespective of the lethality of the virus [17]. Song *et al*. screened host miRNAs and found that miR-323, miR-491 and miR-654 inhibit the replication of the H1N1 IAV in MDCK cells by binding to the 3′ coding region rather than the 3′-UTR of the PB1 gene. Generally, a target mRNA is degraded if the miRNA and the mRNA are a perfect complementary sequence match, whereas translational repression is the most common effect if the miRNA and the mRNA are not a perfect complementary sequence match. However, this previous study showed that the imperfect binding between the miRNAs and PB1 mRNA could down-regulate PB1 expression through mRNA degradation [31]. They explained that the PB1 gene is not of sufficient length to contain miRNA-binding sites in the 3′-UTR sequence and postulated that other mechanisms may play a role in directing the action of miRNAs. In our study, cellular miR-let-7c is a perfect complementary sequence match to the 3′-UTR of the M1 gene and could therefore reduce M1 expression at both the (+) cRNA and protein level. The differences in the results between the Song *et al*. study and our study may derive from the different role and replication process that PB1 and M1 genes have in regulating the IAV life cycle in host cells. Moreover, the A549 cells used in the present study differed from the MDCK cells; the two cell types were derived from different animal species as well as from different tissues. Therefore, it is still worthwhile to investigate the regulation mechanism(s) of cellular miRNA on the IAV genes.

In conclusion, our results indicate that cellular let-7c suppresses IAV replication in host cells by targeting the 3′-UTR of M1 (+) cRNA. The findings suggest that let-7c binding to the 3′-UTR of M1 may serve as a therapeutic target for the prophylaxis and control of influenza virus infections.

## Supporting information

**Fig. S1** Let-7 target site prediction in human cells. Let-7c or let-7a was predicted to pair with residues at the 3′-UTR of the indicated human genes by PITA or TargetScan database screens. MCM5, minichromosome maintenance complex component 5; EIF2AK2, eukaryotic translation initiation factor 2-alpha kinase 2; KPNA1, karyopherin alpha 1; PA2G4, proliferation-associated 2G4.

**Fig. S2** Luciferase reporter assays of let-7c on MCM5, KPNA1, EIF2AK2 and PA2G4 expression in A549 cells. Data were normalized to β-galactosidase activity.

**Fig. S3** Western blotting analysis of caspase-3 expression at 48 hrs post-infection of IAV. Let-7a regulates caspase-3 protein expression but not let-7c.
