# Supplementary material for: Cellular microRNA let-7c inhibits M1 protein expression of the H1N1 influenza A virus in infected human lung epithelial cells
Source: J Cell Mol Med. 2012 Sep 26;16(10):2539–46. doi: 10.1111/j.1582-4934.2012.01572.x (PMC3823446; doi:10.1111/j.1582-4934.2012.01572.x)
Supplement: Supplementary file 1 [file jcmm0016-2539-SD1.doc]

**Supplemental Figures:**

caspase-3 target:


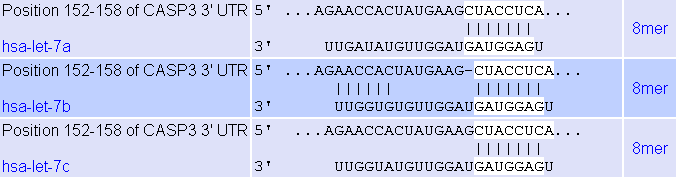

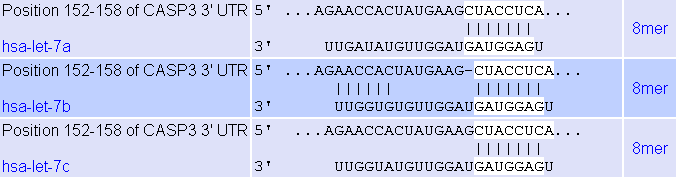


EIF2AK2 target site 1:

EIF2AK2 target site 2:

MCM5 target site 1:

MCM5 target site 2:

PA2G4 target site：

**S-Fig. 1. Let-7 target site prediction in human cells.** Let-7c or let-7a was predicted to pair with residues at the 3′-UTR of the indicated human genes by PITA or TargetScan database screens. MCM5, minichromosome maintenance complex component 5; EIF2AK2, eukaryotic translation initiation factor 2-alpha kinase 2; KPNA1, karyopherin alpha 1; PA2G4, proliferation-associated 2G4.
