# Supplementary material for: Cellular microRNA let-7c inhibits M1 protein expression of the H1N1 influenza A virus in infected human lung epithelial cells
Source: J Cell Mol Med. 2012 Sep 26;16(10):2539–46. doi: 10.1111/j.1582-4934.2012.01572.x (PMC3823446; doi:10.1111/j.1582-4934.2012.01572.x)
Supplement: Supplementary file 2 [file jcmm0016-2539-SD2.docx]

**S-Fig. 2. Luciferase reporter assays of let-7c on MCM5, KPNA1, EIF2AK2, and PA2G4 expression in A549 cells**. Data was normalized to β-galactosidase activity.
