# Supplementary material for: Cellular microRNA let-7c inhibits M1 protein expression of the H1N1 influenza A virus in infected human lung epithelial cells
Source: J Cell Mol Med. 2012 Sep 26;16(10):2539–46. doi: 10.1111/j.1582-4934.2012.01572.x (PMC3823446; doi:10.1111/j.1582-4934.2012.01572.x)
Supplement: Supplementary file 3 [file jcmm0016-2539-SD3.docx]

pcDNA3.1+

let-7a

let-7c

Caspase 3


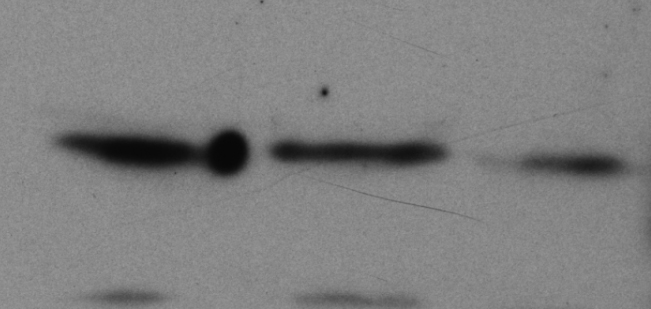


Actin


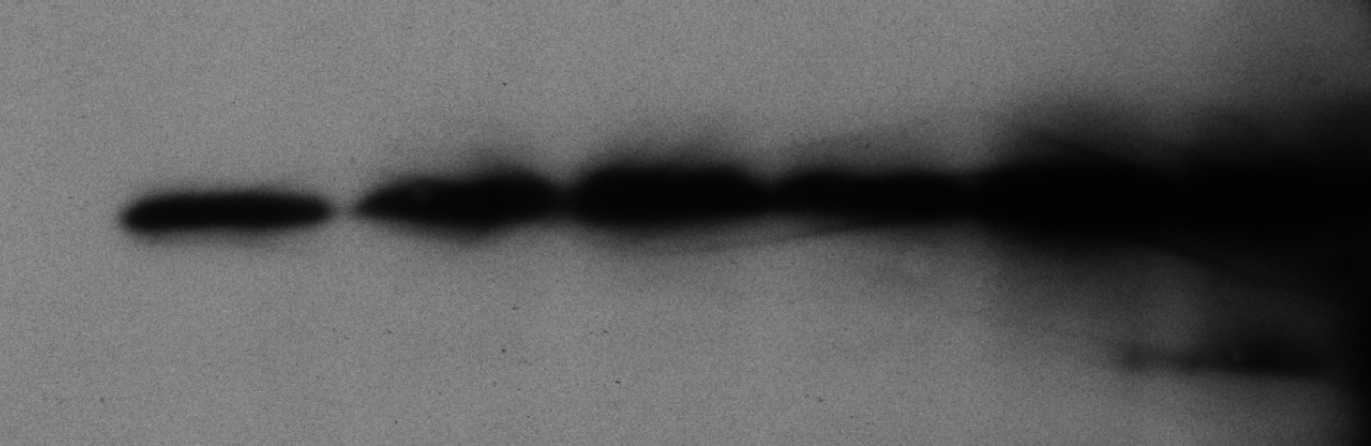


**S-Fig. 3. Western blotting analysis of caspase-3 expression at 48 h post-infection of IAV.** Let-7a regulates caspase-3 protein expression but not let-7c.
